# Milestone achieved: FDA approves nivolumab in combination with cisplatin and gemcitabine for metastatic urothelial carcinoma

**DOI:** 10.1097/MS9.0000000000002601

**Published:** 2024-09-25

**Authors:** Nawal Khaliq, Maryam Sohail, Shanzay Younas, Maryam Ishaq, Aymar Akilimali

**Affiliations:** a Dow University of Health Sciences, Karachi, Pakistan; b D.G. Khan Medical College, Dera Ghazi Khan, Pakistan; c Punjab Medical College, Faisalabad Medical University, Faisalabad, Pakistan; d Khyber Medical College, Peshawar, Khyber Pakhtunkhwa, Pakistan; e Department of Research, Medical Research Circle (MedReC), Goma, Democratic Republic of Congo

## Background

Urothelial carcinoma, also known as transitional cell carcinoma, arises in the specialized epithelium of urinary bladder, urothelium, invades the basement membrane or deeper structures by malignant cells^1^. Based on the recent data, it remains the ninth most common tumor globally which has caused over 213 000 deaths in 2020, worldwide^2,3^. Bladder cancer is highly prevalent and prone to frequent recurrences and progression even after local treatment, resulting in a significant burden on health services^3^. This article aims on Nivolumab + Gemcitabine-Cisplatin, an FDA-approved combination therapy for first-line treatment of advanced and unresectable urothelial carcinoma. It thoroughly explains the treatment’s pharmacokinetics, recommended dosage, mode of action, and the prospective advantages it may offer to urothelial carcinoma patients. The objective of this article is to provide valuable insights into this therapeutic approach, to improve the understanding of the potential benefits of this therapy.

## Pathogenesis

The pathogenesis of urothelial carcinoma is multifactorial, involving gene mutations in TP53, RB1, FGFR3, and HRAS, environmental exposure to tobacco smoke, chronic inflammation resulting from urinary tract infections, and prolonged catheter use, all contributing to tumor progression^4^. This condition may present with symptoms like abdominal pain, hematuria, bone pain or tenderness, fatigue, dysuria, urinary frequency and urgency, urinary incontinence, and weight loss^5^. Early diagnosis and treatment are vital for better outcomes, as bladder cancer can present in two forms: one is the noninvasive forms, which is confined to the bladder’s inner layers, the other is the invasive types that penetrate deeper into the urothelium and have the ability to metastasize^1^.

## Therapeutic approaches

Most of the early, nonmuscle-invasive urothelial carcinoma are treated with transurethral resection of the bladder tumor with intravesical chemotherapy or intravesical Bacillus Calmette-Guerin (BCG) immunotherapy^3^. The muscle-invasive carcinomas can be treated with surgery (cystectomy) followed by adjuvant or neo-adjuvant cisplatin-based chemotherapy, whereas unresectable, locally advanced cancers are given chemotherapy only^3^. Approximately 5% of newly diagnosed bladder cancer patients have metastatic urothelial carcinoma (mUC), which unfortunately, shows limited prognosis, with a 5-year survival rate of less than 5%^3^.

## Current treatment options for metastatic UC

### Platinum-based chemotherapy

For metastatic UC, experts generally recommend the use of platinum-based combination chemotherapies. Patients who do not possess any comorbidity, have adequate renal function are eligible to receive cisplatin-based chemotherapy as the first-line treatment. Gemcitabine plus cisplatin or dose-dense methotrexate, vinblastine, doxorubicin, and cisplatin (MVAC) are the recommended cisplatin-based combination therapies. Those who are not fit to tolerate cisplatin receive carboplatin-based regimens (e.g. gemcitabine plus carboplatin)^3^.

Gemcitabine and cisplatin are commonly used chemotherapy agents for urothelial carcinoma. Gemcitabine works by inhibiting ribonucleotide reductase and impedes DNA synthesis^6^ whereas cisplatin exerts its antineoplastic effects by forming crosslinks with purine bases of DNA, interrupts the DNA repair mechanism, causes DNA damage and ultimately, inducing death of cancer cells^7^. A phase 2, randomized clinical trial studied the efficacy of gemcitabine plus cisplatin and gemcitabine plus carboplatin regimens in patients with metastatic urothelial carcinoma. The results demonstrated improved clinical efficacy with gemcitabine-cisplatin regimens with the overall response (OR) as 49.1% for Gemcitabine-cisplatin (CR: 14.5%; PR: 34.5%) and 40.0% for Gemcitabine-carboplatin (CR: 1.8%; PR: 38.2%). Median time to progression was found to be 8.3 months for Gemcitabine-cisplatin and 7.7 months for Gemcitabine-carboplatin. Median survival rate was 12.8 months and 9.8 months, respectively^8^.

### Targeted therapies

Several studies have assessed targeted therapy when given along with first-line chemotherapeutic agents for advanced urothelial carcinoma^8^. One study evaluated the patient response after administrating gemcitabine-cisplatin with or followed by gefitinib, an inhibitor of epidermal growth factor receptor (EGFR). Patients who received gemcitabine-cisplatin without gefitinib led to a median overall-survival (OS) of 15.9 months, whereas those who received chemotherapy with gefitinib resulted in a median OS of 13.3 months and 8.5 months for gefitinib when administered in sequential treatment^9^.

### Immunotherapy

Immunotherapy in drug agents like pembrolizumab and nivolumab, are immune checkpoint inhibitors (ICI) that target programmed cell death protein 1 and its ligand (PD-1/PD-L1) and heighten T cell-mediated immune response^6^. ICIs have shown better clinical efficacy when assessed by multiple studies in different treatment settings^10^. ICIs are used as switch-maintenance therapy for patients previously treated with platinum-based chemotherapy or for platinum-refractory advanced UC as a second-line therapy^10^. More recently, the phase III double-blind Checkmate 274 clinical trial led to nivolumab becoming the first FDA-approved immune checkpoint inhibitor (ICI) for use in the adjuvant setting for patients with urothelial carcinoma (UC) who have undergone radical surgery and are at high risk of recurrence^10^. Patients with PD-L1 expression, treated with nivolumab as adjuvant therapy, showed better clinical outcomes. Median-disease-free survival (DFS) for nivolumab was found to be 20.8 months as compared to placebo, which had a DFS of 10.8 months^11^.

## Nivolumab plus Gemcitabine-Cisplatin combination therapy

Treating patients with unresectable and locally advanced urothelial carcinoma was a great challenge hence, a new therapy was required which could deal with difficult-to-treat disease to offer better clinical outcomes to patients with a chance to live longer. Till date, no novel agent had demonstrated improved survival outcomes when administered concurrently with platinum-based chemotherapies in the first-line treatment of urothelial carcinoma but Nivolumab, an immune-checkpoint inhibitor, possessed the potential to address the unmet needs of the patients^12^. In phase 3, checkmate 901 clinical trial, nivolumab, when administered with gemcitabine-cisplatin therapy, showed promising clinical efficacy and safety profile outcomes^12^. Based on this clinical trial, FDA-approved nivolumab combination therapy for first-line treatment of advanced and unresectable urothelial carcinoma.

## Key findings of the checkmate 901 trial

The efficacy of nivolumab in combination with cisplatin-gemcitabine was evaluated in Checkmate 901 (NCT03036098) phase 3, a multinational, open-label randomized clinical trial where a total of 608 patients with previously untreated unresectable or metastatic urothelial carcinoma were randomized^12^. These patients were divided into two groups (304 patients in each group) who either received intravenous combination of nivolumab (at a dose of 360 mg) plus gemcitabine-cisplatin every 3 weeks for up to six cycles, followed by nivolumab (at a dose of 480 mg) every 4 weeks for a maximum of 2 years or received gemcitabine-cisplatin alone every 3 weeks for up to six cycles^12^.

At a median follow-up of 33.6 months, the group treated with nivolumab combination therapy showed overall survival (OS) of 21.7 months and progression-free survival (PFS) of 7.9 months, which is significantly longer as compared to OS of 18.9 months and PFS of 7.6 months with gemcitabine-cisplatin alone. The overall objective response was significantly improved with nivolumab-combination therapy than gemcitabine-cisplatin alone. Objective response was reported as 57.6 and 43.1%, respectively^12^. Another outcome, complete response (CR) was also studied. A complete response of 21.7% was achieved with nivolumab combination therapy and 11.8% was seen with gemcitabine-cisplatin alone (Table 1)^12^.

**Table 1:** Efficacy of Nivolumab + Gemcitabine-Cisplatin and Gemcitabine-Cisplatin

Variables	Nivolumab + Gemcitabine-Cisplatin	Gemcitabine-Cisplatin
Overall survival	21.7 months	18.9 months
Progression-free survival	7.9 months	7.6 months
Overall objective response	57.6%	43.1%
Complete response	21.7%	11.8%
Median-therapy duration	7.4 months	3.7 months

This trial also assessed patient’s health-related quality of life from baseline through week 16, after administrating the treatment. Quality-of-life was evaluated using European Organization for Research and Treatment of Cancer Quality of Life Questionnaire–Core 30 (EORTC QLQ-C30) Global Health Status score. The results were found to be stable in both the groups with a change of not more than 10 points up to week 16^12^.

## Recommended dosage and side effects

Nivolumab is recommended as a first-line treatment for adult patients with metastatic or invasive urothelial carcinoma (mUC) in conjunction with cisplatin and gemcitabine for a maximum of six cycles, each cycle lasts for 21 days^13^. On day 1, 360 mg of intravenous nivolumab is given, along with 70 mg/m² of cisplatin and 1000 mg/m² of gemcitabine, which is administered on days 1 and 8^14^. Following up to six cycles, nivolumab 240 mg IV every 2 weeks or 480 mg every 4 weeks is given as a single agent until the illness progresses, the toxicity is intolerable, or for a maximum treatment period of 2 years from the first dose^14^.

In the safety analysis as performed in checkmate 901 clinical trial, 304 patients in the nivolumab-combination group and 288 patients in the gemcitabine-cisplatin group were included^12^. The median therapy duration was 7.4 months for the nivolumab-combination group and 3.7 months for the gemcitabine-cisplatin group^12^. Adverse events due to any cause were observed in 99.7% of the nivolumab-combination group and 98.6% of the gemcitabine-cisplatin group; grade 3 or higher adverse events occurred in 76.6 and 67.7% of patients, respectively. There was one grade 5 treatment-related adverse event (sepsis) in the nivolumab-combination group and one (acute kidney injury) in the gemcitabine-cisplatin group^12^. Treatment-related adverse events of any grade leading to discontinuation were reported in 21.1% of the nivolumab-combination group and 17.4% of the gemcitabine-cisplatin group^12^. Immune-mediated adverse events of any grade occurred in 89 patients receiving nivolumab combination therapy and in two patients on gemcitabine-cisplatin therapy alone (Table 2)^12^.

**Table 2:** Adverse Effects of Nivolumab + Gemcitabine-Cisplatin and Gemcitabine-Cisplatin

Adverse effects	Nivolumab + Gemcitabine-Cisplatin	Gemcitabine-Cisplatin
Adverse events of any cause	99.7%	98.6%
Adverse events leading to discontinuation	21.1%	17.4%
Grade 3 or higher events	76.6%	67.7%
Immune-mediated events	Eighty-nine patients	Two patients

## Future perspectives

The treatment options for metastatic bladder cancer is rapidly evolving, with variations in access to new therapies worldwide, leading to diverse use of nivolumab, gemcitabine, and cisplatin combinations. While combination chemotherapy remains an ideal regimen, immunotherapy, antibody-drug conjugates (ADCs), and tyrosine kinase inhibitors (TKIs) are also gaining prominence, particularly in early-stage urothelial cancer. Advances in screening technologies, like transcriptomics, metabolomics, and proteomics, have made target biomarkers identification a key research priority. Discovering specific markers of bladder cancer stem cells (BCSCs) could lead to the development of targeted therapies, addressing the significant drug resistance often seen with chemotherapy. Designing clinical trials that combine therapies targeting various pathways, including signaling and metabolic pathways, holds promise for improving survival outcomes in advanced bladder cancer^15^.

Nivolumab with platinum-doublet chemotherapy presents a comprehensive safety profile that will help healthcare providers and patients understand the risks involved and make informed decisions about the treatment. Patients mostly present with manageable side effects like rash, vomiting, nausea, fatigue, musculoskeletal discomfort, and constipation^14^. Any organ system or tissue may have immune-mediated adverse reactions, which can be severe or lethal. These immune-mediated conditions include pneumonitis, colitis, hepatitis/hepatotoxicity, and nephritic adverse effects^14^. Proper and timely diagnosis and management of immune-mediated symptoms is essential to optimize treatment outcomes while maintaining safety standards^16^. It may also harm the fetus; therefore, warn women about the possibility of pregnancy and the need to use appropriate contraception^14^.

## Conclusion

In conclusion, the FDA’s approval of nivolumab in combination with gemcitabine and cisplatin represents a significant achievement in the first-line treatment of metastatic or unresectable urothelial carcinoma. This combination therapy offers the potential for improved treatment outcomes and extended remission in cancer patients and provides clinically meaningful benefits than gemcitabine-cisplatin therapy alone, in patients with previously untreated metastatic urothelial carcinoma. However, this combination therapy also carries a risk of immune-mediated adverse reactions and other side effects like rash, vomiting, nausea, and musculoskeletal discomfort. Careful consideration and close monitoring are essential to maximize benefits and minimize risks for patients undergoing this treatment regimen.

## Ethical approval

Not applicable.

## Consent

Not applicable.

## Source of funding

This research did not receive any specific grant from funding agencies in the public, commercial, or not-for-profit sectors.

## Author contribution

N.K.: conceptualization, project administration, supervision, and visualization; N.K., M.S., S.Y., and M.I.; writing – original draft; A.A.: writing – review and editing.

## Conflicts of interest disclosure

The authors declare no potential conflicts of interest related to financial, consultant, institutional, authorship, and/or publication of this article.

## Research registration unique identifying number (UIN)

1. Name of the registry: not applicable.
2. Unique identifying number or registration ID: not applicable.
3. Hyperlink to your specific registration (must be publicly accessible and will be checked): not applicable.

## Guarantor

Aymar Akilimali.

## Data availability statement

Not applicable.

## Provenance and peer review

Not commissioned, externally peer-reviewed.
